# Maryland Veterinary Medical Society

**Published:** 1895-02

**Authors:** A. W. Clement

**Affiliations:** Secretary


Secretary.

### MARYLAND VETERINARY MEDICAL SOCIETY.

THE annual meeting was held on Tuesday, February 12th, at the Hotel Studio, in Baltimore. There was a good attendance present, and reports of several cases were discussed. Drs. Dougherty, Hoffman, and Clement reported cases of flatulent colic, requiring the use of the trocar in which the introduction of the canula was followed by the escape of clear fluid, with very little flatus. In each of the cases discussed the animals recovered. A satisfactory explanation of the escape of fluid in such quantities was not arrived at. Dr. Dougherty reported several cases of distention of the guttural pouch, which were relieved by a novel and simple operation, the use of the trocar and canula. Remarkable success had attended this operation in his hands.[1]

Dr. Martinet, of Baltimore, was unanimously reëlected president; Dr. F. H. Macken, of Cecil, vice-president, and Dr. A. W. Clement, of Baltimore, secretary and treasurer.

An enjoyable supper concluded this very interesting meeting.

A. W. Clement,
Secretary.

[1] This paper will appear in the March number.

